# Determinants of dietary patterns in school going adolescents in Urban Zambia

**DOI:** 10.3389/fnut.2022.956109

**Published:** 2022-08-25

**Authors:** Mulenga Mary Mukanu, Peter Delobelle, Anne Marie Thow, Zandile June-Rose Mchiza

**Affiliations:** ^1^School of Public Health, University of the Western Cape, Cape Town, South Africa; ^2^Chronic Disease Initiative for Africa, University of Cape Town, Cape Town, South Africa; ^3^Department of Public Health, Vrije Universiteit Brussel, Brussel, Belgium; ^4^Menzies Centre for Health Policy and Economics, University of Sydney, Camperdown, NSW, Australia; ^5^Non-communicable Diseases Research Unit, South African Medical Research Council, Cape Town, South Africa

**Keywords:** food environment, Zambia, school nutrition, nutrition policy, adolescents

## Abstract

**Background:**

Understanding dietary patterns in a population is critical for decision making. This study aimed to identify the prevailing dietary patterns and their associated individual and school environment factors among school going adolescents in Lusaka, Zambia.

**Method:**

A cross-sectional study involving 404 Grade 10 pupils from 10 secondary schools in Lusaka district was conducted. A 108-item unquantified Food Frequency Questionnaire (FFQ) was used to assess the learner's food intake practices. Principal component analysis (PCA) was used to derive dietary patterns from the 108 food items. In addition, a mapping of food vendors and types of food sold was conducted in the same 10 schools using a semi-structured observation checklist. Bivariate and multivariate multilevel regression was used to analyse the individual and school level determinants of the adolescent dietary patterns.

**Results:**

The average age of learners was 16.1 years (SD 1.4 years); 234 (58%) were female while 170 (42%) male. “Snacking,” “vegetarian,” “health conscious,” and “traditional” dietary patterns accounting for 54.5% of variability in learner's diets were identified using PCA. At individual level, having weekly pocket money was significantly associated with snacking (*p* ≤ 0.0001). Self-identified poverty was associated with snacking (*p* ≤ 0.0001), vegetarian (*p* = 0.009) and traditional (*p* = 0.009) dietary patterns. School level factors like a school tuckshop (similar to canteen) that sells fast foods or a *kantemba (semi-permanent makeshift store)* within the school vicinity (*p* = 0.023) were significantly associated with a snacking dietary pattern. School tuckshop selling *nshima (a thick maize based porridge)* was significantly associated with vegetarian (*p* = 0.007), health conscious (*p* = 0.02) and traditional dietary patterns (p=0.01) while a tuckshop with fruit significantly predicted traditional (*p* ≤ 0.0001), vegetarian (*p* = 0.041), and snacking (*p* = 0.002), dietary patterns. Having a supermarket or fast food restaurants in the school vicinity did not significantly influence any dietary pattern.

**Conclusion:**

Both individual behavioral and school environment level factors were found to be significant determinants of the four dietary patterns identified in this study.

## Introduction

Dietary practices have drastically shifted in Low- and Middle-Income Countries (LMICs) in recent years (1, 2). Most countries are experiencing the nutrition transition and dealing with the double burden of malnutrition (3). Understanding prevailing dietary practices in a population is critical for decision making as it provides insights into short term and long-term diseases outcomes in the population (4). Consequently, there is a growing interest in understanding the dietary practices of adolescents as it represents one of the key intervention points in the lifecycle of a person (5). The adolescence period provides an opportunity for cost effective policy action and investments that can help establish healthy dietary patterns that reduce the risk of nutrition related health outcomes like obesity, diabetes and hypertension later in adulthood (6, 7).

Dietary pattern analysis is one of the methods used to profile the nutrition status of a population (8). The term dietary pattern refers to the quantities, proportions, variety, or combination of different foods, drinks, and nutrients in diets, and the frequency with which they are habitually consumed (9). The dietary pattern analysis approach has gained popularity over more traditional methods like single food or nutrient analysis because of the recognition that no single nutrients are consumed in isolation but are part of a larger diet (10). Studying the whole diet therefore provides a better way to explore correlations between different dietary components and the interactions between diet and health.

Adolescents are generally associated with the snacking dietary pattern, characterized by consumption of snacks, sugar sweetened drinks, and fast foods (11). Demographic factors like age and gender as well as social economic status (SES) factors like income (12) are among the well-known individual level influencers of dietary patterns in adolescents. The school food environment is another important factor that influences adolescents' dietary patterns as adolescents spend a significant amount of time in school. The school structure provides an opportunity for adolescents to express their autonomy through independent food choices (13). Healthy dietary patterns characterized by frequent consumption of fruit and vegetables has been associated with availability of these food options in school tuck-shops (14). Conversely, availability of fast food outlets within school vicinities has been associated with unhealthy dietary patterns (15, 16).

While there is a growing body of knowledge on adolescent dietary patterns and their determinants from LMICs (17–20), this area remains understudied in Zambia. Adolescent nutrition assessments have largely focused on micronutrient deficiencies in girls of child bearing age (21, 22). Moreover, other assessments with a nutrition component like the WHO STEPS survey only include people above 18 years (23), under-representing adolescents who make up a quarter of Zambia's population (24). The Global School Health Survey which generates nutrition related data on school going children was last conducted in 2004 (25). This study therefore aims to identify the prevailing dietary patterns and the individual and school environment factors associated with dietary patterns among school going adolescents. The study will generate evidence that can guide decisions on nutrition policy reforms that might be required to support healthier dietary patterns among school going adolescents in urban settings.

## Materials and methods

### Study design

A cross sectional survey of school going adolescents in selected secondary schools in Lusaka District was conducted in order to establish their dietary patterns and to map the food environment surrounding the schools. The survey involved administration of a food frequency questionnaire (FFQ) to school going adolescents in 10 sampled schools.

### Study sites

The study was conducted in ten purposively sampled secondary schools in in Lusaka District of Lusaka, the capital city of Zambia. At the time of the research, Lusaka District had fifty-four secondary schools (seven private-owned and forty-one government) registered with the Ministry of General Education.

### Study population

The survey was administered to randomly selected, consenting Grade 10 pupils in the sampled schools. Only Grade 10 learners (both male and female) in the selected schools participated in the study, because this class is a non-exam class and participation would hence cause minimum disruption of the adolescent's school activity. In addition, it is expected that Grade 10 pupils (on average aged between 14 and 16 years) would have well developed food preferences to accurately complete the questionnaire (26).

### Study tools

We administered a three-part questionnaire to collect data used to determine the dietary patterns of the adolescents. The first part of the questionnaire collected socio-demographic data including age, sex, residence, and self-assessed poverty. The second part was adapted from the nutritional behavior module of the global school health survey (27) and included questions assessing nutrition related practices such as consumption of fruit and vegetables or snacks and exposure to fast food advertisements in the previous 30 days. The third part was the unquantified food frequency questionnaire (FFQ) which was developed based on the Food and Agriculture Organization (FAO)'s dietary assessment guide for low resource settings (8). The 108 food items included in the FFQ in this study were adapted from the Zambian food guidelines produced by the National Food and Nutrition Commission (NFNC) (28). The questions in the FFQ asked learners to rate how often they consumed a food item in the previous month on an ordinal scale of never; seldom (less than once a month); 1–3 times per month; 1–2 times per week; 3–4 times per week; or daily.

A semi-structured observation checklist was developed and used to map typology of vendors and food sold around the school environment. The food types were categorized into seven main groups: (1) fruits; (2) snacks (such as biscuits, potato crisps, fritters, doughnuts, roasted groundnuts, roasted maize); (3) fast foods (including fried chicken, sausage, pizza, pies, chips, samosas); (4) soft drinks (including carbonated sugar sweetened drinks and energy drinks); (5) water; (6) milk and (7) *nshima* (a thick maize based porridge eaten with relish). Food vendors were categorized as either school tuckshops, street vendors, *kantemba* (a semi-permanent makeshift store), supermarket, grocery or convenience store.

Construct validity of the FFQ, including face and content validity was assessed by a nutrition expert from the NFNC. Both tools were pilot tested among 40 learners in two schools with similar characteristics to the schools included in the study sample before data collection. The purpose of the pilot testing was to assess the readability, logic of questions and length of interviews. Based on feedback from the pilot test, the FFQ was revised to include local names of some food items. The data collection tools were not translated into the local languages, but administered in English, Bemba or Nyanja by trained research assistants.

### Sampling

Ten secondary schools, representing 20% of the total population of secondary schools in Lusaka District were purposively selected for the research. The 10 schools (nine government owned and one private owned) were purposively selected to ensure a representation of various demographics and socio-economic status of adolescents in Lusaka. Purposive sampling based on the residential area where a school was located was used as proxy for socio-economic status of adolescents. This is based on studies that have shown an inverse relationship between population density of a residential area and economic status (29). Data was collected from a total of 404 adolescents in the ten sampled secondary schools based on calculation of the minimum sample size for infinite populations (with a 95% confidence level and +/−5% precision) (30).

### Recruitment and data collection

The lead author sought and obtained permission first from the Ministry of General Education and then from individual school administrators before the study was conducted. Data was collected over a period of 2 days for each school. On the first day, the trained research assistant obtained the sampling frame, consisting of the names of all grade 10 learners (available on that day^1^), from the teacher assigned by the school administrator to oversee the study once the school granted permission to proceed with the research. The lead author then entered the sampling frame into Stata and assigned each learner a unique random number. The random numbers were then sorted in ascending order and the first 40 learners on the list were invited to participate in the study. Where a learner declined to participate, a replacement was made with the next learner on the list. The selected learners were given information sheets and parent permission forms for their guardian to consent to their participation. On the second day, all learners with parental permission where further asked to provide assent to participate in the research by completing an assent form. Learners were informed that they still had the right to choose not to participate in the research with no negative consequences for them. The FFQ was only administered to learners who assented and had parental permission.

Data was collected in November 2020. The FFQ and school food environment checklist were programmed into electronic versions using Open Data Kit for ease of data collection. Data was collected by five research assistants who were trained and supervised by the lead author. All research assistants had a minimum of a bachelor's degree, at least 1-year experience collecting quantitative data and native speakers of Bemba or Nyanja, the main local languages used in Lusaka. The research assistants were trained on ethical conduct of research where justice, beneficence and right of person was emphasized. Research assistant were also trained on dietary assessment in adolescents using a FFQ and how to administer the questions in both English, Bemba and Nyanja. All interviews were conducted within the school premises after class.

### Data analysis

Data analysis was done in three steps for each type of data from the different sections of the tool as described below. All data analyses were conducted in Stata version 15 (31).

#### Step 1: Analysis of demographic, nutritional behavior, and school environment

Descriptive statistics were used to summarize the categorical demographic and nutritional behavior data (see Table 1) as well as school environment data on food and vendors types (Table 2) into proportions or frequencies. In addition, food intake variables were summarized into three categories of frequency of consumption (Supplementary Table 1).

**Table 1:** Survey participant characteristics (*n* = 404).

**Characteristic**	**Frequency**	**Percentage (%)**
**Gender**		
Female	234	58
Male	170	42
**Age**		
14–16	268	67
17 and above	136	33
**Self-assessed poverty**		
Poor	83	21
Non-poor	321	79
**Guardian employed**		
Employed	321	79
Unemployed	83	21
**Had weekly pocket money**		
Yes	303	75
No	101	25
**Household size**		
Up to 5	158	39
6–10	234	58
More than 10	12	3
**Residence**		
High density	220	54
Medium density	109	27
Low density	75	19
**Eat breakfast at home**		
Always	170	42
Regularly	164	41
Rarely	55	13
Never	15	4
**Eat breakfast at school**		
Always	11	3
Regularly	16	4
Rarely	6	1
Never	371	92
**Eat lunch at school**		
Always	12	3
Regularly	18	4
Rarely	8	2
Never	366	91
**Saw TV advertisement for fast foods (*****n*** **=** **389)**		
Always	104	27
Regularly	247	63
Rarely	34	9
Never	4	1
**Bought sugar sweetened beverage at school**		
Yes	247	39
No	157	61
**Bought fast food at school**		
Yes	233	58
No	171	42
**Healthy eating taught at school (*****n*** **=** **374)**		
Yes	235	63
No	139	37
**Benefits of fruit and vegetables taught at school (*****n*** **=** **379)**		
Yes	248	65
No	131	35

**Table 2:** Food environment characteristics of the schools (*n* = 10).

**Characteristic**	**Frequency**	**Percentage**
**Tuck shop present**		
Yes	10	100
**Types of food sold in the school tuck shop**		
**Snacks**		
Yes	10	100
No		
**Fruit**		
Yes	1	10
No	9	90
**Fast foods**		
Yes	3	30
No	7	70
***Nshima***		
Yes	2	20
No	8	80
**Sugar sweetened beverages**		
Yes	10	100
No		
**Milk**		
Yes	1	10
No	9	90
**Water**		
Yes	10	100
No		
**Food sold by vendors around school (*****n*** **=** **10)**		
**Snacks**		
Yes	100	100
No		
**Fruit**		
Yes	9	90
No	1	10
**Fast food**		
Yes	7	70
No	3	30
***Nshima***		
Yes	6	60
No	4	40
**Sugar sweetened beverages**		
Yes	10	100
No		
**Milk**		
Yes	8	80
No	2	20
**Water**		
Yes	10	100
No		
**Types of vendors around school (*****n*** **=** **10)**		
**Supermarket**		
Yes	2	20
No	8	80
***Kantemba***		
Yes	5	50
No	5	50
**Street vendors**		
Yes	10	100
No		
**Grocery**		
Yes	6	60
No	4	40
**Fast food restaurant**		
Yes	5	50
No	5	50

#### Step 2: Analysis of dietary patterns

Principal component analysis (PCA) was used to obtain a dietary pattern for each adolescent in the study based on data from the food intake variables. Principal component analysis is an exploratory data driven approach that uses a correlation matrix of food intake variables to identify common patterns of food consumption within the data to account for the largest amount of variation in diet [i.e., statistical technique to determine dietary patterns based on shared variance across dietary variables].

The reduction of food intake variables into food groups was done in three steps. In the first step, the food intake variables, which were measured on an ordinal scale, were recoded and converted to frequency of consumption per week. The conversion of variables was done as follows: “never” = 0; “seldom (less than once a month)” = 0; “1–3 times per month” = 0.5; “1–2 times per week” = 1.5; “3–4 times per week” = 3.5; and “daily” = 7. In the second step, all the food intake variables were allocated to one of the 15 broad food groups based on the FAO/WHO Global Individual Food consumption data Tool (GIFT) nutrition-sensitive food grouping (see Supplementary Table 2) (32). In the last step, a compound score for the food group was obtained by summing the individual scores of the food intake variables in each food group. A higher compound score for a food group indicated a higher frequency of consumption of the food items under that food group.

Principal component analysis was then performed on the 15 food groups to identify correlations that point to particular dietary patterns. The Kaiser–Meyer–Olkin (KMO) test, a measure of sampling adequacy used to assess the suitability of the data for PCA retained an overall value of 0.84 warranting a factor analysis. Varimax (orthogonal) rotation was performed following PCA to improve the interpretability of results. Orthogonal rotation following PCA is useful as it maximizes loadings of variables on extracted factors while minimizing loading on other factors. Four components were retained using the scree plot (Supplementary Figure 1) and Kaiser's stopping rule (33) of an eigenvalue of ≥1, which has been applied in other studies (12, 20). The four components accounted for 54.6% of total variance in food intake among the school going adolescents (see Supplementary Table 3). A cut off point of >0.30 on the factor loading was used to identify significant food groups in each component and to determine which dietary pattern best described the component.

#### Step 3: Analysis of determinants of identified dietary patterns

Multilevel regression analysis was conducted to determine the statistically significant determinants of the dietary patterns identified in the PCA of food groups. This analysis method addressed the possible non-independence in the sample which might result in clustering of learner behaviors at school level. An intra-cluster correlation (ICC) analysis was conducted using a cut off level of 0.05 to confirm the presence of clustering and the need for a multilevel model.

The dependent variables in the multilevel model were the four dietary patterns represented by the components retained from the PCA. Predictor variables were classified into two groups. The first group contained demographic characteristics such as age, gender, and household size. The second group included variables related to the food environment, such as presence of a tuck shop at school, exposure to food marketing and types of food vendors around a school. An investigator led step wise approach was used to fit the model. In the first step, a bivariate regression between each identified dietary pattern and predictor variables was conducted to identify significant predictors with a 95% confidence interval (**Table 4**). This was followed by fitting of the full model containing both the individual level and food environment predictors. The model with the least ICC and without any multicollinearity was retained and reported in **Table 5**.

### Ethics considerations

Ethics clearance for the study was obtained from the University of the Western Cape's Humanities and Social Sciences Research Ethics Committee (HSSREC) in South Africa (reference number HS20/6/19) and Eres Converge ethic review board in Zambia [reference number 2020-Aug-012). Parental consent was sought for learners to participate in the study and individual assent obtained from learners.

## Results

### Participant characteristics

Table 1 summarizes the demographic characteristics of the learners who completed the food frequency questionnaire. A total of 404 students were interviewed of whom 234 (58%) were female and 170 (42%) male. The average age of learners was 16.1 years (SD 1.4 years).

Analysis of SES characteristics showed that most students identified as being non-poor (79%); had a guardian who was employed (79%) and had weekly pocket money (75%) averaging K38 (US$2). A little over half of the students (54%) were from high-density neighborhoods and the most common household size was 6–10 people (58%).

In terms of dietary patterns, most students reported that they always or regularly ate breakfast (80%); were not on a diet (80%); and did not eat breakfast (91%) or lunch (92%) at school. More students said they bought fast foods (58%) than sugar sweetened beverages (39%) at school.

A similar proportion of students reported learning about healthy eating (63%) and benefits of eating fruit and vegetables (65%) at school.

### Description of the school food environment

Table 2 summarizes the characteristics of the school environment for the 10 secondary schools. All schools had a tuckshop within the school premises. While snacks, SSBs and water were sold in 100% of the tuckshops, only a few schools had tuckshops selling fruit (10%), milk (10%), and *nshima* (20%). The most common type of food vendors outside the school premises was street vendors who were present around all of the schools. Groceries were found around 60% of the schools while fast food restaurants and *kantemba* were present outside 50% of the schools. Only 20% of the schools had a supermarket within walking distance of the school premises.

### Description of the identified dietary patterns

The factor loading of the 15 food groups for the four retained components from the PCA are presented in Table 3. Based on these factor loadings, we identified 4 main dietary patterns as prevalent among the students and these are described below. The name for each dietary pattern was chosen to represent the food groups with high factor loadings accounting for the most significant variation in that dietary pattern. The first component explained 29.9 % variance; the three remaining components explained 8.8, 8.5, and 7.3% of the variance in food intake, respectively.

**Table 3:** Factor loadings of the 15 food groups in the four principal components extracted from the PCA.

**Variable**	**Comp1: Snacking** **(29.9%)**	**Comp2: Vegetarian** **(8.8%)**	**Comp3: Health conscious** **(8.5%)**	**Comp4: Traditional** **(7.3%)**
Cereal	−0.0547	0.0947	0.1100	**0.6172**
Roots	0.0264	**0.3530**	−0.0830	0.2755
Pulses	0.0187	**0.4591**	0.0923	−0.0124
Milk	**0.3671**	−0.1337	0.1892	0.1604
Fish	−0.0545	**0.5662**	−0.1786	0.1389
Meat	0.2609	−0.0714	−0.0032	**0.4658**
Insect	0.2643	0.0276	−0.0536	**−0.3309**
Vegetables	0.0061	**0.4570**	0.2257	−0.2035
Fruit	0.1536	0.1745	**0.4144**	−0.2615
Fats	**0.3452**	−0.0179	−0.0335	0.1723
Sweets	**0.4611**	−0.1464	−0.0131	0.0008
Soft drink	**0.3584**	0.1913	−0.2254	−0.0233
Beverage	0.2119	0.0478	**0.3284**	0.0050
Savory snacks	**0.4322**	0.0936	−0.1027	−0.1452
Egg products	−0.0695	−0.0514	**0.7133**	0.1019

The bold values indicate the food groups with factor loading above the cut off point of >0.30, which significantly contribute to the dietary pattern identified.

#### “Snacking” dietary pattern

Component 1 was named “snacking” dietary pattern because it had high positive factor loadings for snacks, sweets, soft drinks, fats and milk. The most commonly consumed food items in the snacks group were *jiggies (processed corn-based snacks)* (59%), potato chips (20%), and potato crisps (15%). Fritters and sweets were the most consumed food items in the sweets group with over a quarter of adolescents saying they eat them at least three times a week. Ice-cream, chocolate and cake were relatively less frequently consumed. In the soft drinks group, locally produced products like *freezits (sugary beverage sold frozen)* (64%) and various brands of carbonated SSBs (22%) were frequently consumed compared to international brands like Coca-Cola (14%).

#### “Vegetarian” dietary pattern

Component 2 was named “vegetarian” pattern as it had high positive factor loadings for roots, pulses, fish and vegetables. In the pulses food group, dried beans (14%) was the most commonly consumed legume followed by soya (11%). Modern vegetables like rape (64%), cabbage (15%), and cucumber (17%) and more traditional vegetables like pumpkin leaves (21%), sweet potato leaves (19%), and okra (15%) were most frequently consumed in the vegetable group. Dried *kapenta (small sardine like fish)* (17%) followed by fresh fish was the most frequently consumed food in the fish and shellfish group.

#### “Healthy conscious” dietary pattern

Component 3 was named “health conscious” because of the high positive factor loading on fruits and eggs, and negative factor loadings on snacks, soft drinks, sweets, and fats. Bananas (41%), oranges (19%) and apples (16%) compared to local or home-grown fruits like watermelon (14%), guava (8%), and pineapples (5%) were frequently consumed in the fruits group. Nearly almost half of the adolescents reported they consume eggs three times a week or more and as many as 80% consume them at least once a week.

#### Traditional dietary pattern

Component 4 was named “traditional” based on high factor loadings for cereals and meat and negative factor loadings on fruit, vegetables and snacks. The majority of adolescents (80%) said they eat breakfast meal *nshima* three times a week or more compared to roller meal *nshima* (23%). Chicken was the most common meat protein consumed with two-thirds of the adolescents reporting they ate it at least once a week. Small livestock like rabbit and goal were rarely consumed.

### Determinants/drivers of dietary patterns

Table 4 shows the results of the bivariate analysis of the dependent variables and the individual and school environment level predictors while Table 5 shows the results of multilevel analysis of the fitted model.

**Table 4:** Bivariate analysis of the individual and food environment predictors of dietary patterns of adolescents.

**Predictor**	**Snacking**			**Vegetarian**			**Healthy**			**Traditional**		
	**Mean pattern score**	***P*-value**	**95% CI**	**Mean pattern score**	***P*-value**	**95% CI**	**Mean pattern score**	***P*-value**	**95% CI**	**Mean pattern score**	***P*-value**	**95% CI**
**Individual level predictors**												
Gender												
Female	1			1			1			1		
Male	−0.526	**0.002**	−0.860, −0.192	−0.103	0.423	−0.357, 0.150	−0.112	0.366	−0.356, 0.131	0.059	0.580	−0.150, 0.268
Age												
14–16	1			1			1			1		
17 and above	−0.412	**0.020**	−0.765, −0.060	0.082	0.544	−0.184, 0.345	−0.024	0.857	−0.279, 0.232	−0.078	0.489	−0.297, 0.142
Guardian employed	−0.014	0.944	−0.421, 0.392	−0.317	**0.040**	−0.620, −0.014	0.194	0.194	−0.099, 0.488	0.222	0.082	−0.028, 0.473
Poor	−1.053	**0.0001**	−1.449, −0.657	−0.360	**0.020**	−0.664, −0.056	−0.375	**0.012**	−0.668, −0.083	−0.434	**0.001**	−0.684, −0.185
Had weekly income	0.006	**0.0001**	0.003, 0.009	0.001	0.645	−0.002, 0.003	0.002	0.174	−0.001, 0.004	−0.001	0.506	−0.003, 0.001
Eating breakfast at home												
Always	1			1			1			1		
Regularly	−0.274	0.140	−0.637, 0.090	0.679	0.628	−0.206, 0.342	−0.123	0.356	−0.385, 0.138	−0.364	**0.001**	−0.586, −0.142
Rarely	−0.417	0.129	−0.956, 0.121	−0.053	0.798	−0.461, 0.354	−0.055	0.780	−0.440, 0.330	−0.372	**0.027**	−0.702, −0.042
Never	−0.744	0.099	−1.628, 0.139	−0.243	0.473	−0.908, 0.421	−0.835	**0.010**	−1.470, −0.196	−0.936	**0.001**	−1.474, −0.397
Saw fast food adverts on TV												
Always	1			1			1			1		
Regularly	−0.183	0.342	−0.560, 0.194	−0.209	0.142	−0.487, 0.070	−0.088	0.527	−0.361, 0.185	−0.098	0.407	−0.329, 0.133
Rarely	−0.536	0.103	−1.182, 0.109	−0.428	0.079	−0.906, 0.050	0.113	0.635	−0.353, 0.578	−0.150	0.458	−0.547, 0.247
Never	−0.841	0.316	−2.486, 0.803	−0.054	0.931	−1.271, 1.163	1.011	0.095	−0.177, 2.201	0.450	0.382	−0.560, 1.459
Benefits of fruit and vegetables taught	0.063	0.731	−0.298, 0.425	−0.006	0.966	−0.277, 0.265	−0.017	0.896	−0.278, 0.243	0.158	0.164	−0.065, 0.381
**School food environment predictors**												
School tuck shop sells *nshima*	0.060	0.917	−1.063, 1.183	−0.857	0.142	−2.005, 0.288	−0.364	0.250	−0.984, 0.257	0.065	0.905	−1.000, 1.130
School tuck shop sells fruit	1.039	0.119	−0.266, 2.345	1.194	0.112	−0.277, 2.668	0.624	0.102	−0.125, 1.372	0.436	0.535	−0.943, 1.816
School tuckshop sells milk	−0.541	0.451	−0.866, 1.949	−0.377	0.651	−2.001, 1.255	−0.550	0.150	−1.299, 0.198	1.606	**0.001**	0.640, 2.573
Vendor sells snacks	−0.816	0.114	−1.828, 0.196	−0.400	0.535	−1.665, 0.864	0.014	0.965	−0.630, 0.658	−0.873	**0.042**	−1.717, −0.030
Vendor sells fruit	−0.816	0.114	−1.828, 0.196	−0.400	0.535	−1.665, 0.864	0.014	0.965	−0.630, 0.658	−0.873	**0.042**	−1.717, −0.030
Vendor sells *nshima*	−0.007	0.987	−0.892, 0.877	0.866	0.051	−0.004, 1.737	−0.001	0.996	−0.512, 0.510	0.318	0.407	−0.434, 1.071
Vendor sells SSB	−0.816	0.114	−1.828, 0.196	−0.400	0.535	−1.665, 0.864	0.014	0.965	−0.630, 0.658	−0.873	**0.042**	−1.717, −0.030
Vendor sells fast foods	−0.481	0.278	−1.351, 0.389	−0.166	0.754	−1.201, 0.869	−0.297	0.244	−0.796, 0.202	0.568	0.129	−0.166, 1.301
Vendor sells milk	−0.685	0.131	−1.573, 0.203	−0.024	0.967	−1.140–1.092	0.185	0.508	−0.362, 0.731	−0.932	**0.005**	−1.589, −0.277
Vendor sells water	−0.816	0.114	−1.828, 0.196	−0.400	0.535	−1.665, 0.864	0.014	0.965	−0.630, 0.658	−0.873	**0.042**	−1.717, −0.030
Fast food restaurant	0.133	0.768	−0.748, 1.013	0.479	0.328	−0.480, 1.438	0.189	0.458	−0.309, 0.687	−0.125	0.751	−0.894, 0.645
Grocery	0.308	0.486	−0.559, 1.176	−0.364	0.466	−1.344, 0.615	0.339	0.161	−0.136, 0.815	−0.331	0.386	−1.079, 0.417
*Kantemba*	−0.474	0.273	−1.322, 0.373	−0.498	0.310	−1.460, 0.464	0.059	0.822	−0.453, 0.570	−0.673	**0.048**	−1.341, −0.005
Street vendor	−1.035	0.138	−2.403, 0.333	−1.215	0.127	−2.778, 0.347	−0.573	0.154	−1.361, 0.215	0.025	0.970	−1.302, 1.352
Supermarket	−0.192	0.736	−1.314, 0.929	0.946	0.110	−0.214, 2.105	0.170	0.604	−0.472, 0.812	0.152	0.762	−0.837, 1.142

Predictors with a p < 0.05 were considered significant. The bold values represent the significant predictors of the diaetary patterns at 95% confidence level.

**Table 5:** Multivariate analysis of the individual and food environment predictors of dietary patterns of adolescents.

**Predictor**	**Snacking**			**Vegetarian**			**Healthy**			**Traditional**		
	**Mean pattern score**	***P*-value**	**95% CI**	**Mean pattern score**	***P*-value**	**95% CI**	**Mean pattern score**	***P*-value**	**95% CI**	**Mean pattern score**	***P*-value**	**95% CI**
**Individual level predictors**												
Gender												
Female	1			1			1			1		
Male	−0.285	0.157	−0.678, 0.109	−0.017	0.913	−0.325, 0.291	−0.036	0.812	−0.336, 0.263	0.112	0.366	−0.131, 0.354
Age												
14–16	1			1			1			1		
17 and above	−0.225	0.279	−0.634, 0.183	0.229	0.161	−0.091, 0.549	0.047	0.768	−0.264, 0.357	0.036	0.777	−0.215, 0.287
Guardian employed	−0.639	**0.006**	−1.097, −0.182	−0.617	**0.001**	−0.977, −0.256	0.055	0.757	−0.293, 0.403	0.011	0.938	−0.270, 0.292
Poor	−0.679	**0.009**	−1.186, −0.172	−0.511	**0.012**	−0.911, −0.112	−0.101	0.609	−0.486, 0.285	−0.412	**0.010**	−0.724, −0.100
Had weekly income	0.004	**0.01**	0.001, 0.008	0.0001	0.889	−0.003, 0.002	0.001	0.291	−0.001, 0.004	−0.002	**0.029**	−0.004, 0.0001
Eating breakfast at home												
Always	1			1			1			1		
Regularly	−0.575	**0.005**	−0.981, −0.169	−0.037	0.822	−0.358, 0.284	−0.256	0.103	−0.565, 0.052	−0.428	**0.001**	−0.677, −0.179
Rarely	−0.628	**0.035**	−1.213, −0.043	−0.226	0.344	−0.696, 0.243	−0.145	0.523	−0.589, 0.300	−0.312	0.088	−0.671, 0.046
Never	−0.526	0.279	−1.479, 0.426	−0.052	0.891	−0.801, 0.697	−0.899	**0.015**	−1.624, −0.175	−0.860	**0.004**	−1.446, −0.275
Saw fast food adverts on TV												
Always	1			1			1			1		
Regularly	−0.285	0.169	−0.690, 0.121	−0.288	0.075	−0.605, 0.029	−0.079	0.616	−0.388, 0.230	−0.175	0.171	−0.425, 0.075
Rarely	−0.646	0.059	−1.318, 0.025	−0.554	**0.039**	−1.081, −0.027	0.192	0.462	−0.319, 0.702	−0.291	0.167	−0.704, 0.122
Never	−1.113	0.18	−2.740, 0.514	−0.148	0.82	−1.420, 1.125	1.008	0.110	−0.230, 2.245	0.724	0.157	−0.278, 1.726
Benefits of fruit and vegetables taught	0.245	0.228	−0.153, 0.643	0.084	0.598	−0.228, 0.396	0.055	0.722	−0.248, 0.358	0.163	0.193	−0.082, 0.408
**School food environment predictors**												
School tuck shop sells fruit	1.747	**0.002**	0.637, 2.857	1.332	**0.041**	0.053, 2.610	0.443	0.296	−0.389, 1.276	1.333	**<0.0001**	0.689, 1.976
School tuck shop sells *nshima*	0.003	0.992	−0.561, 0.566	−0.884	**0.007**	−1.524, −0.244	−0.502	**0.020**	−0.924, −0.079	0.431	**0.010**	0.103, 0.759
School tuckshop sells SSB	−0.449	0.262	−1.233, 0.335	0.616	0.201	−0.327, 1.559	0.650	**0.030**	0.063, 1.236	−1.398	**<0.0001**	−1.848, −0.948
School tuckshop sells fast food	−0.656	**0.025**	−1.232, −0.080	−0.492	0.162	−1.182, 0.198	−0.278	0.206	−0.708, 0.152	0.064	0.702	−0.265, 0.394
Supermarket	−0.687	0.107	−1.521, 0.148	0.191	0.700	−0.780, 1.162	−0.248	0.437	−0.873, 0.377	−0.372	0.131	−0.854, 0.111
*Kantemba*	−0.719	**0.023**	−1.338, −0.101	−0.902	**0.012**	−1.605, −0.198	0.051	0.831	−0.414, 0.515	−0.578	**0.002**	−0.938, −0.218
Fast food restaurant	0.095	0.779	−0.567, 0.756	−0.027	0.944	−0.783, 0.729	0.046	0.856	−0.450, 0.542	0.052	0.79	−0.332, 0.436

Predictors with a p < 0.05 were considered significant. The bold values represent the significant predictors of the diaetary patterns at 95% confidence level.

#### Individual-Level predictors of dietary patterns

From the bivariate analysis, being male (*p* = 0.002) and being above 17 years were significant predictors of snacking dietary pattern. Being poor significantly predicted all the dietary patterns while having a guardian who is employed (*p* = 0.040) and having pocket money (*p* ≤ 0.0001) predicted vegetarian and snacking dietary patterns, respectively. Eating breakfast at home also predicted traditional dietary pattern.

From the multivariate model, SES and dietary habit variables were found to be significant predictors of dietary patterns. Pocket money was a significant predictor of the snacking dietary pattern: a higher amount of weekly pocket money was associated with a higher factor score and a high likelihood for the snacking dietary pattern (*p* = 0.01). Paradoxically, students who self-identified as poor were also likely to have a snacking dietary pattern (*p* ≤ 0.0001) as well as vegetarian (*p* = 0.009) and traditional (*p* = 0.009) dietary pattern. In addition, vegetarian dietary pattern was significantly predicted by exposure to food advertisements: adolescents who rarely saw adverts for fast food and drinks on TV were more like to have a vegetarian dietary pattern compared to those who saw such adverts frequently. Neither age, gender nor being taught about benefits of eating fruit and vegetables significantly predicted any dietary pattern.

#### School-Level predictors of dietary patterns

From the bivariate analysis, we found that none of the school level predictors significantly influenced the snacking, vegetarian and healthy dietary patterns. However, having a school tuckshop sells milk (0.001), vendor that sells milk (0.005), and *kantemba* (*P* = 0.045) predicted the traditional dietary pattern.

Results from the multivariate analysis showed the snacking dietary pattern was significantly predicted by having a tuckshop that sells fast foods within the school and the presence of a *Kantemba* (*p* = 0.023) within the school vicinity. A school tuckshop selling *nshima* significantly predicted vegetarian (*p* = 0.007), health conscious (*p* = 0.02) and traditional dietary patterns (*p* = 0.01) while a tuckshop with fruit significantly predicted traditional (*p* ≤ 0.0001), vegetarian (*p* = 0.041), and snacking (*p* = 0.002), dietary patterns. Having a supermarket or fast food restaurants in the school vicinity did not significantly influence any dietary pattern.

## Discussion

This study assessed the dietary patterns of school going adolescents in urban Zambia as well as the individual and school-level determinants of these patterns. Four main dietary patterns were identified as most prevalent among the adolescents: (1) snacking pattern characterized by consumption of snacks and sweets; (2) vegetarian characterized by consumption of pulses, fish and vegetables; (3) health conscious characterized by fruits and eggs; and (4) traditional characterized by consumption of cereals and meat. Individual level SES factors like having weekly pocket money, a guardian who is employed and self-assessed poverty as well as school environment factors like having a tuckshop that sells fruits or *nshima* and having street vendors outside the school were significant predictors of the identified dietary pattern.

The snacking dietary pattern identified in this study is common and has been demonstrated in school going adolescents in developing countries (34). The predominance of the snacking dietary pattern is evidence of the ongoing nutrition transition toward more westernized diets which features fast foods, snacks and sweets, and is associated with development of cardiometabolic conditions (35). Interestingly, while the focus of nutrition advocates has largely been on the role of “big food” in the dual burden of malnutrition (36, 37), the snacking dietary pattern observed in our study was characterized by consumption of locally manufactured brands of biscuits, carbonated drinks and sweets and less of international brands. This observed difference could be because locally produced snacks are relatively cheaper than international brands. These findings highlight the need for a contextualized approach that account for country specific nuances like locally manufactured unhealthy foods in finding solutions for addressing the dual burden of malnutrition. For instance, the Zambia government has prioritized the food and beverage subsector which currently makes up 65% of the local manufacturing industry as a key driver of economic growth through job creation (38). It would be worthwhile for the government to also incentivize healthy food production so that the economic needs are balanced with promoting population health.

This study found that the relatively healthier and less common vegetarian and healthy conscious dietary patterns were also present among adolescents. The vegetarian dietary pattern in our study was characterized by high factor loadings on roots, pulses, fish, and vegetables. Consumption of similar food groups by vegetarian adolescents has been demonstrated in other contexts (39, 40). Vegetarian dietary patterns are associated with better long-term health outcomes like lower cardiovascular risk scores (41) and better bone structure (42), and should thus be promoted among adolescents. However, vegetarian dietary pattern has also been associated with negative behaviors such as eating and weight disorders among adolescents in high income countries (43). As the vegetarian dietary pattern is relatively uncommon in Zambia, additional qualitative research using phenomenological designs might be required to understand the factors associated with this dietary pattern to better inform interventions aimed at promoting uptake of such diets among adolescents.

Dietary patterns termed “traditional” are usually country specific, consisting of indigenous foods and country specific staples (19, 20, 44–46). Similarly, the traditional dietary pattern identified in this study was characterized by high factor loadings on cereals and meat, exemplifying the typical Zambian meal that consists of *nshima* and protein and/or relish. The persistence of consumption of traditional foods like *nshima* and vegetables associated with the vegetarian and traditional dietary patterns presents an opportunity to promote preservation of indigenous foods that has potential to contribute to slowing the nutrition transition.

Socio-economic factors like income have consistently been shown to influence dietary patterns (47). Studies from high- and middle-income countries suggest that unhealthy food consumption is relatively higher in low income populations compared to those with higher incomes (48, 49). In developing countries, evidence shows an inverse relationship between SES and unhealthy food consumption (12). Similarly, this study found a significant association between SES and dietary patterns, but the effects were observed across all dietary patterns. On one hand, adolescents who had weekly pocket money were more likely to exhibit a snacking and traditional dietary pattern while those who had an employed guardian likely had a snacking and vegetarian dietary pattern. On the other hand, self-assessed poverty significantly predicted snacking, vegetarian and traditional dietary patterns.

Unhealthy dietary patterns have been linked to lack of nutrition-related knowledge (50). For instance, a study in rural Italy found an association between nutrition knowledge scores and the number of snacks a student consumed in a day (51). Generally, adolescents in our study had a good nutrition knowledge with 75% reporting having “been taught” about the benefits of fruits and vegetables. However, having knowledge on the benefits of fruit and vegetable consumption did not significantly influence any dietary pattern in our study population. While we did not assess the accuracy of the self-reported nutrition-related knowledge, there might be need to evaluate the method used to educate adolescents on nutrition to identify how knowledge can translate to better dietary habits. This finding also supports the need for additional non-informational policy measures to promote healthier dietary patterns. Information-based interventions have been widely used for addressing nutrition related problems owing to their ease of implementation and relative lack of resistance from stakeholders (52). Despite their popularity, evidence of the impact of information-based interventions on nutrition behavior change is mixed (53). However, information-based interventions like mass campaigns remain valuable for raising public awareness in support of more effective, more contentious policy options like taxations and restrictions (54, 55).

The school food environment is an important influencer of dietary patterns as it determines the proximity, availability and cost of different food options (56). School tuckshops and semi-permanent food vendors were significantly associated with adolescents' dietary patterns in our study population compared to supermarkets and fast food restaurants, which have been identified in other studies (14–16). One reason for this finding might be that supermarkets are cost prohibitive for adolescents who our findings showed only have on average $2 dollar per week of pocket money. Our findings suggest that more informal semi-permanent vendors are key stakeholders in promoting healthy dietary patterns in urban school environments as they had a higher food variety and healthier food options compared to school tuckshops and were likely to be more affordable than supermarkets. Similar findings linking informal food vendors to increased fruit and vegetable consumption have been shown in Tanzania (57) and the US (58).

This study found that school environment level factors in addition to individual factors were significant determinants of dietary patterns in adolescents. Currently, school nutrition interventions as outlined in the school health and nutrition policy largely focus on behavior change of adolescents through educational measures (59). However, our findings imply that there is an urgent need for policy measures that will promote healthier school environments. The government in Zambia should consider implementing policies that have been shown to be effective for the school going adolescents such as healthy food provisioning policies, school food meals and school food standards (60). These policy measures should be adapted to make them relevant for the Zambian context.

This study used PCA to determine dietary patterns of school going adolescents in urban Zambia from data collected using a FFQ. Data from FFQs have been associated with recall bias because it is dependent on a respondent's memory to report what they ate over a long period of time (10). The self-reported measures of poverty and income that were used as measures of SES are also associated with social desirability bias (61). In addition, PCA does not provide information about the actual quantities consumed for each food group in each pattern (62). Therefore, these findings should be interpreted with these limitations in mind. Despite this, the large sample size and high response rate contribute to the strength of the findings. Moreover, the approach of using PCA with FFQ data has been shown to be reliable and widely used to study dietary patterns of a specific population (63, 64). Our findings therefore remain useful as they have provided insight into existing dietary patterns of school going adolescents in Zambia, a very under-researched population, and are essential for developing public health interventions. Because dietary patterns are highly context specific, the generalizability of our findings is limited to other urban settings similar to Lusaka. Future research is required to: (1) determine the dietary patterns and food environments in rural settings and (2) analyse how the different dietary patterns correlated with nutritional outcomes like body mass index which can be used to estimate risk for adverse health outcomes like diabetes and hypertension.

## Conclusion

Dietary behavior among school going adolescents in Urban Lusaka was found to be characterized by four dominant dietary patterns in this study: snacking, vegetarian, health-conscious and traditional. Individual level predictors of these dietary patterns included socio-economic characteristics such as having a guardian who was employed and an adolescent having pocket money or self-assessed poverty. School environment characteristics such as having a tuckshop that sold fruits or *nshima* and having street vendors and *kantemba* outside the school vicinity were also associated with the four identified dietary patterns. The significance of both individual behavioral and school environment level factors in influencing dietary patterns in this context points to the need for solutions that go beyond behavior change based educational interventions if Zambia is to promote healthy food consumption patterns among its school-going adolescents.

## Data availability statement

The raw data supporting the conclusions of this article will be made available by the authors, without undue reservation.

## Ethics statement

The studies involving human participants were reviewed and approved by ERES Converge (2020-Aug-012) in Zambia and the University of the Western Cape's Humanities and Social Sciences Research Ethics Committee (HSSREC) in South Africa (Reference Number: HS20/6/19). Participation in the study was voluntary. Written informed consent to participate in this study was provided by the participants' legal guardian/next of kin. All the participants below 18 years provided written assent before the could be enrolled in the study.

## Author contributions

MM: conceptualization, formal analysis, investigation, and writing—original draft preparation. MM, AT, PD, and ZM methodology and writing—review and editing. AT, PD, and ZM validation. All authors contributed to the article and approved the submitted version.

## Conflict of interest

The authors declare that the research was conducted in the absence of any commercial or financial relationships that could be construed as a potential conflict of interest.

## Publisher's note

All claims expressed in this article are solely those of the authors and do not necessarily represent those of their affiliated organizations, or those of the publisher, the editors and the reviewers. Any product that may be evaluated in this article, or claim that may be made by its manufacturer, is not guaranteed or endorsed by the publisher.

## Footnotes

^1^Because of the COVID-19 prevention measures, we found that schools had randomly split classes to report on different days in order to maintain social distancing.
