# Supplementary material for: Determinants of dietary patterns in school going adolescents in Urban Zambia
Source: Front Nutr. 2022 Aug 25;9:956109. doi: 10.3389/fnut.2022.956109 (PMC9452662; doi:10.3389/fnut.2022.956109)
Supplement: Supplementary file 1 [file Data_Sheet_1.docx]

# SUPPLEMENTARY TABLES

Supplementary table 1: Frequency of consumption of food items under each food group

| **Food item** | **Food group** | **3 times a week or more (%)** | **Once a week or more (%)** |
| --- | --- | --- | --- |
| *Munkoyo* | Beverage | 16 | 32 |
| *Chibwantu* | Beverage | 13 | 28 |
| Breakfast meal *nshima* | Cereals and their products | 80 | 83 |
| Bread | Cereals and their products | 75 | 88 |
| Rice | Cereals and their products | 34 | 73 |
| Roller meal *nshima* | Cereals and their products | 23 | 26 |
| Pasta | Cereals and their products | 18 | 52 |
| Samp | Cereals and their products | 7 | 21 |
| Millet | Cereals and their products | 1 | 3 |
| Sorghum | Cereals and their products | 0 | 3 |
| Cassava flour | Cereals and their products | 3 | 8 |
| Eggs | Eggs | 48 | 79 |
| Cooking oil | Fats and oils | 52 | 81 |
| Margarine | Fats and oils | 24 | 56 |
| Butter | Fats and oils | 15 | 21 |
| Palm oil | Fats and oils | 0 | 1 |
| Dried *kapenta* | Fish, shellfish and their products | 17 | 50 |
| Fresh fish | Fish, shellfish and their products | 13 | 36 |
| Dry fish | Fish, shellfish and their products | 9 | 26 |
| Fresh *kapenta* | Fish, shellfish and their products | 2 | 17 |
| Banana | Fruits and their products | 31 | 60 |
| Mango | Fruits and their products | 30 | 41 |
| Orange | Fruits and their products | 19 | 50 |
| Apple | Fruits and their products | 16 | 41 |
| Watermelon | Fruits and their products | 14 | 31 |
| Lemon | Fruits and their products | 13 | 28 |
| Guava | Fruits and their products | 8 | 17 |
| Pineapple | Fruits and their products | 5 | 17 |
| *Masau* | Fruits and their products | 3 | 9 |
| *Masuku* | Fruits and their products | 3 | 6 |
| Grapes | Fruits and their products | 3 | 13 |
| Strawberry | Fruits and their products | 2 | 5 |
| Pawpaw | Fruits and their products | 2 | 9 |
| Mulberry | Fruits and their products | 2 | 5 |
| Tamarind | Fruits and their products | 1 | 3 |
| *Tungai* | Fruits and their products | 1 | 3 |
| Baobab fruit | Fruits and their products | 1 | 4 |
| Pure fruit juice | Fruits and their products | 2 | 7 |
| Caterpillar | Insects, grubs and their products | 2 | 9 |
| Grasshoppers | Insects, grubs and their products | 0 | 0 |
| Crickets | Insects, grubs and their products | 0 | 0 |
| Termites | Insects, grubs and their products | 0 | 1 |
| Chicken | Meat and meat products | 24 | 62 |
| Beef | Meat and meat products | 16 | 47 |
| Pork | Meat and meat products | 4 | 19 |
| Game meat | Meat and meat products | 2 | 5 |
| Goat meat | Meat and meat products | 2 | 15 |
| Rabbit meat | Meat and meat products | 0 | 0 |
| Fresh milk | Milk and milk products | 33 | 54 |
| Powered milk | Milk and milk products | 14 | 28 |
| Sour milk | Milk and milk products | 9 | 29 |
| Ice-cream | Milk and milk products | 8 | 27 |
| Yoghurt | Milk and milk products | 6 | 23 |
| Cheese | Milk and milk products | 4 | 8 |
| Condensed milk | Milk and milk products | 3 | 6 |
| Goat milk | Milk and milk products | 0 | 1 |
| Soy milk | Milk and milk products | 1 | 3 |
| Ground nuts (Paste or powder) | Pulses, seeds and nuts and their products | 20 | 37 |
| Roasted groundnuts | Pulses, seeds and nuts and their products | 14 | 34 |
| Dried beans | Pulses, seeds and nuts and their products | 14 | 52 |
| Soya | Pulses, seeds and nuts and their products | 11 | 27 |
| Peas | Pulses, seeds and nuts and their products | 2 | 7 |
| Cowpea | Pulses, seeds and nuts and their products | 0 | 1 |
| Pumpkin seed | Pulses, seeds and nuts and their products | 0 | 1 |
| Irish potatoes | Roots, tubers, plantains and their products | 24 | 58 |
| Fresh cassava | Roots, tubers, plantains and their products | 9 | 13 |
| Dried roasted cassava | Roots, tubers, plantains and their products | 7 | 17 |
| *Chikanda* | Roots, tubers, plantains and their products | 6 | 16 |
| Sweet potatoes | Roots, tubers, plantains and their products | 6 | 11 |
| Yam | Roots, tubers, plantains and their products | 1 | 1 |
| *Busala* | Roots, tubers, plantains and their products | 0 | 3 |
| Jiggies | Savoury snacks | 59 | 77 |
| Fritter | Savoury snacks | 27 | 57 |
| Chips | Savoury snacks | 20 | 51 |
| Crisps | Savoury snacks | 15 | 42 |
| *Freezit* | Soft drinks | 64 | 82 |
| Local sugar sweetened beverage | Soft drinks | 22 | 49 |
| Coca cola/Fanta/Sprite | Soft drinks | 14 | 35 |
| Energy drink | Soft drinks | 7 | 19 |
| Sweets | Sweets and sugar | 25 | 41 |
| Chocolate | Sweets and sugar | 7 | 16 |
| Cake | Sweets and sugar | 4 | 11 |
| Rape | Vegetables and their products | 64 | 91 |
| Green pepper | Vegetables and their products | 28 | 48 |
| Pumpkin leaves | Vegetables and their products | 21 | 56 |
| Sweet potato leaves | Vegetables and their products | 19 | 55 |
| Cucumber | Vegetables and their products | 17 | 42 |
| Carrot | Vegetables and their products | 16 | 35 |
| Okra | Vegetables and their products | 15 | 42 |
| Cabbage | Vegetables and their products | 15 | 54 |
| Green beans | Vegetables and their products | 13 | 36 |
| *Bondwe* | Vegetables and their products | 12 | 32 |
| Spinach | Vegetables and their products | 10 | 27 |
| *Impwa* | Vegetables and their products | 7 | 35 |
| *Lumanda* | Vegetables and their products | 7 | 22 |
| Cassava leaves | Vegetables and their products | 4 | 13 |
| Lettuce | Vegetables and their products | 4 | 12 |
| Bean leaves | Vegetables and their products | 3 | 13 |
| Other traditional vegetables | Vegetables and their products | 2 | 6 |
| Mushroom | Vegetables and their products | 1 | 3 |
| Broccoli | Vegetables and their products | 1 | 4 |
| Cowpea leaves | Vegetables and their products | 0 | 1 |
| Wild mushroom | Vegetables and their products | 0 | 1 |
| *Lumanda* | Vegetables and their products | 5 | 8 |

Supplementary table 2: Food grouping based on WHO/FAO list

| **Food group** | **Food item included** |
| --- | --- |
| 1. Cereals and their products | Breakfast meal *nshima*, roller meal *nshima,* rice, samp, pasta, bread, millet, sorghum |
| 1. Roots, tubers, plantains and their products | Potatoes, fresh cassava, dry cassava, sweet potatoes, *busala, chikanda*, yam |
| 1. Pulses, seeds and nuts and their products | Cowpeas, beans, soya, groundnuts, peas, pumpkin seed, soy milk, roast groundnuts |
| 1. Milk and milk products | Fresh milk, sour milk, yoghurt, cheese, goat milk, powdered milk, condensed milk, ice cream |
| 1. Eggs and their products | Eggs |
| 1. Fish, shellfish and their products | Fresh fish, dry *kapenta*, fresh *kapenta*, dry fish |
| 1. Meat and meat products | Chicken, beef, pork, goat, game, rabbit |
| 1. Insects, grubs and their products | Grasshopper, crickets, caterpillars, termites |
| 1. Vegetables and their products | Pumpkin, mushroom, potato leaves, pumpkin leaves, bondwe, okra, bean leaves, cassava leaves, cowpea leaves, *impwa, lumanda*, green beans, lettuce, green pepper, broccoli, carrot, spinach, cabbage, rape, cucumber |
| 1. Fruits and their products | Banana, orange, pineapple, apple, grapes, strawberry, watermelon, lemon, mango, guava, pawpaw, mulberry, tamarind, *tungai*, *masau*, *chibuyu*, *Masuku*, mango juice, orange juice, fruit juice |
| 1. Fats and oils | Margarine, cooking oil, butter, palm oil |
| 1. Sweets and sugars | Chocolates, cake, sweets, biscuits |
| 1. Beverages | *Munkoyo, chibwantu* |
| 1. Soft drinks | Coca cola, sugar sweetened carbonated beverage, energy drinks, *freezit* |
| 1. Savoury snacks | Chips, crisps, *jiggies*, fritters |
|  |  |

Supplementary Table 3: Eigen values for the dietary patterns/components

| **Component** | **Eigenvalue** | **Difference** | **Proportion** | **Cumulative** |
| --- | --- | --- | --- | --- |
| Comp1 | **4.48966** | 3.15549 | 0.2993 | 0.2993 |
| Comp2 | **1.33417** | 0.0521565 | 0.0889 | 0.3883 |
| Comp3 | **1.28202** | 0.190501 | 0.0855 | 0.4737 |
| Comp4 | **1.09152** | 0.162312 | 0.0728 | 0.5465 |
| Comp5 | 0.929206 | 0.124946 | 0.0619 | 0.6084 |
| Comp6 | 0.80426 | 0.011434 | 0.0536 | 0.6621 |
| Comp7 | 0.792826 | 0.0859983 | 0.0529 | 0.7149 |
| Comp8 | 0.706827 | 0.0302105 | 0.0471 | 0.762 |
| Comp9 | 0.676617 | 0.0767968 | 0.0451 | 0.8071 |
| Comp10 | 0.59982 | 0.0256588 | 0.04 | 0.8471 |
| Comp11 | 0.574161 | 0.0749153 | 0.0383 | 0.8854 |
| Comp12 | 0.499246 | 0.0500842 | 0.0333 | 0.9187 |
| Comp13 | 0.449162 | 0.0355088 | 0.0299 | 0.9486 |
| Comp14 | 0.413653 | 0.0568016 | 0.0276 | 0.9762 |
| Comp15 | 0.356851 | . | 0.0238 | 1 |

Figure 1: Scree plot of the eigenvalues
